# Impact of wearable physical activity monitoring devices with exercise prescription or advice in the maintenance phase of cardiac rehabilitation: systematic review and meta-analysis

**DOI:** 10.1186/s13102-019-0126-8

**Published:** 2019-07-30

**Authors:** Amanda L. Hannan, Michael P. Harders, Wayne Hing, Mike Climstein, Jeff S. Coombes, James Furness

**Affiliations:** 1 Faculty of Health Sciences and Medicine, Bond University, 2 Promethean Way, Robina, Qld, Gold Coast, Queensland 4226 Australia; 2 Physical Activity, Lifestyle, Ageing and Wellbeing Faculty Research Group Faculty of Health Sciences, University of Sydney, Lidcombe, NSW Australia; 3 School of Human Movement and Nutrition Sciences, The University of Queensland, Brisbane, Australia; 4 School of Health and Human Sciences, Southern Cross University, Gold Coast, QLD Australia

**Keywords:** Exercise, Cardiac rehabilitation, Maintenance phase, Cardiovascular disease, Coronary artery disease, Wearable devices

## Abstract

**Background:**

Physical activity (PA) is a component of cardiac rehabilitation (CR). However, life-long engagement in PA is required to maintain benefits gained. Wearable PA monitoring devices (WPAM) are thought to increase PA. There appear to be no reviews which investigate the effect of WPAM in cardiac populations. We firstly aimed to systematically review randomised controlled trials within the cardiac population that investigated the effect WPAM had through the maintenance phase of CR. We specifically examined the effect on cardiorespiratory fitness (CRF), amount and intensity of daily PA, and sedentary time. Secondly, we aimed to collate outcome measures reported, reasons for drop out, adverse events, and psychological impact from utilising a WPAM.

**Methods:**

A systematic search (up to January 2019) of relevant databases was completed, followed by a narrative synthesis, meta-analysis and qualitative analysis.

**Results:**

Nine studies involving 1,352 participants were included. CRF was improved to a greater extent in participants using WPAM with exercise prescription or advice compared with controls (MD 1.65 mL/kg/min;95% confidence interval [CI; 0.64–2.66]; *p* = 0.001; I^2^ = 0%). There was no significant between group difference in six-minute walk test distance. In 70% of studies, step count was greater in participants using a WPAM with exercise prescription or advice, however the overall effect was not significant (SMD 0.45;95% [CI; − 0.17-1.07] *p* = 0.15; I^2^ = 81%). A sensitivity analysis resulted in significantly greater step counts in participants using a WPAM with exercise prescription or advice and reduced the heterogeneity from 81 to 0% (SMD 0.78;95% [CI;0.54–1.02]; *p* < 0.001; I^2^ = 0%). Three out of four studies reporting on intensity, found significantly increased time spent in moderate and moderate-vigorous intensity PA. No difference between groups was found for sedentary time. Three of six studies reported improved psychological benefits.

No cardiac adverse events related to physical activity were reported and 62% of non-cardiac adverse events were primarily musculoskeletal injuries. Reasons for dropping out included medical conditions, lack of motivation, loss of interest, and technical difficulties.

**Conclusions:**

Our meta-analysis showed WPAM with exercise prescription or advice are superior to no device in improving CRF in the maintenance phase of CR and no cardiac adverse events were reported with WPAM use. Our qualitative analysis showed evidence in favour of WPAM with exercise prescription or advice for both CRF and step count. WPAM with exercise prescription or advice did not change sedentary time. Psychological health and exercise intensity may potentially be enhanced by WPAM with exercise prescription or advice, however further research would strengthen this conclusion.

**Trial registration:**

PROSPERO Registration Number: CRD42019106591.

## Background

Deaths from cardiovascular disease (CVD) have risen by 14.5% globally between 2006 and 2016 [1]. A systematic analysis for the Global Burden of Disease, which analysed 264 causes of mortality in 195 locations between 1980 and 2016, reported CVD as being responsible for 17.6 million deaths, of which 85.1% were attributed to coronary heart disease (CHD) and stroke. Deaths attributed to CHD alone rose 19% to 9.48 million during the same period. Additionally, the analysis reported CHD as being the leading cause of years of life lost in 113 countries for men and 97 countries for women [1].

For those who have suffered a myocardial infarction, the risk of subsequent cardiovascular events within 5 years increases by 20% [2]. Globally, secondary prevention guidelines and action plans have been developed to combat this healthcare burden [3–5]. For people diagnosed with cardiac disease, attending cardiac rehabilitation (CR) is recommended to aid secondary prevention [4–8]. Cardiac rehabilitation utilises a multidisciplinary approach to improve health through education, risk factor reduction, lifestyle behaviour modification, psychosocial strategies and rehabilitative exercise programs [5–9]. Cardiac rehabilitation is usually delivered across three phases: phase 1 (inpatient setting), phase 2 (outpatient setting) and, phase 3 (maintenance) [5].

Physical activity (PA) is an essential component of CR [5]. Physical activity is any physical movement that requires the expenditure of energy above resting requirements [10]. The exercise component of CR aims to improve the physical functioning (cardiorespiratory fitness (CRF), muscular strength and flexibility) of participants. Cardiorespiratory fitness is defined as the maximum rate of oxygen consumption of the heart, lungs and skeletal muscle during exercise [10]. It has been shown to be inversely proportionate to mortality and predicts prognosis in patients with CHD [11–13]. Research has shown every metabolic equivalent increase in CRF results in a 13–17% reduction in cardiovascular and all-cause mortality [11–13]. Additionally, Martin et al. [14] specifically showed a 13% decrease in overall mortality for every MET increase in CRF following 12 weeks of CR. In addition, each MET increase was associated with a 25%-point reduction in all-cause mortality, for those who maintained CRF gains at 1 year [14].

A recent systematic review and meta-analysis focusing on exercise-based CR in Phase 2, which included 22 studies with 4,834 participants, found the exercise model currently being used, although reducing hospital admissions, had no effect on all-cause mortality [15]. This suggests the CRF gains achieved in CR must, therefore, be maintained long-term to offer a potential reduction in mortality. This is further supported by studies reporting the deleterious effects of physical inactivity [16–18]. Large reductions in daily step count over a two-week period, significantly decreases CRF, insulin sensitivity and lower limb muscle mass whilst increasing body fat, liver fat and LDL cholesterol [18].

Several countries have reported one fifth to one third of eligible patients enrol in CR [19–22]. Australia has reported a higher enrolment rate (51–80%) [23]. To improve this low uptake, researchers have investigated alternate models of CR delivery. A systematic review by Clarke et al. [24] identified 83 studies describing alternate ways of providing CR. These studies were based primarily in Phase 2 CR. They included multifactorial individualised telehealth, internet-based delivery, telehealth interventions focused on exercise, telehealth interventions focused on recovery, community or home-based CR, rural and remote programs and multiple models of care and alternative, complimentary models. The authors concluded that community or home-based CR produce similar reductions in cardiovascular risk factors compared with hospital-based programs. Furthermore, a meta-analysis by Clark et al. [24] found home based programs are an effective and low-cost alternative to hospital-based CR.

In contrast to the numerous studies conducted specific to Phase 2 of CR, there are few studies investigating PA in Phase 3 [25–29]. Of those, Reid et al. [28] found participants did not maintain increased exercise levels beyond 2 months post discharge from Phase 2 CR. Furthermore, Bock et al. [25] reported that only 56% of patients were meeting exercise guidelines at 12 months post-discharge from CR. However, Bock et al. [25] also showed those who participated in a Phase 3 program were significantly more likely to continue regular and more vigorous activity.

A systematic review and meta-analysis published by Claes et al. [26] investigated the longer-term effects of home-based exercise in CHD patients compared with usual care or centre based rehabilitation. Seven studies were included in the meta-analysis on exercise capacity. Results showed no significant differences in exercise capacity between home based and usual care. However, they also found a significant difference in exercise capacity in favour of home-based exercise when compared with centre-based exercise, of small effect size (SMD 0.25, 95% CI 0.02–0.48). Therefore, encouraging life-long PA for patients with CHD at home seems a feasible option to maintain CRF and therefore, potentially reduce mortality.

Activity trackers are worn by over 10% of adults [30] and wearable technology was named number three in the top twenty worldwide fitness trends in 2018 [31]. Wearable technology is thought to improve the amount of, and adherence to, PA [32–35]. A 2016 systematic review identifying 13 randomised controlled trials (RCTs) and 6 quasi-experimental studies utilising a pedometer, found 79% of trials were effective in increasing PA [36]. However, a review by Coughlin et al. [37] to determine the efficacy of wearables in improving PA concluded that larger studies with greater sample sizes, coupled with longer durations, are required to fully support the adoption of WPAM with exercise prescription or advice to increase PA in healthy populations.

Previous research within the CHD population found lack of motivation and time were the most common barriers cited to engaging in PA [38]. This was further supported by Bravata et al. [39] who concluded lack of motivation negatively influenced self-efficacy for exercise. Studies investigating exercise monitoring in the home of people diagnosed with CHD have used various monitoring devices from pedometers through to electrocardiographic transmission [29, 40–49]. There is conflicting evidence of the benefits of WPAM in the CHD population. A systematic review by Bravata et al. [39] found the use of a pedometer significantly increased PA. Similarly, a study by Butler et al. [48] also found that pedometers increased adherence and PA in patients with CHD. In contrast, an earlier study by Butler et al. [47] found no difference in the amount of walking completed by participants wearing a pedometer displaying the step counts, compared to the step counts being obscured from patients. To the authors’ knowledge there appear to be no systematic reviews of RCTs that have investigated the effect of WPAM on the maintenance of PA and CRF/physical capacity in phase 3 CR. Furthermore, no systematic review has collated CRF outcome measures, reasons for dropouts or adverse events in studies investigating WPAM in the CHD population.

We firstly aimed to systematically review randomised controlled trials within the cardiac population that investigated the effect WPAM with exercise prescription or advice had through the maintenance phase of CR. We specifically examined the effect on cardiorespiratory fitness (CRF), amount and intensity of daily PA, and sedentary time. Secondly, we aimed to collate outcome measures reported, reasons for drop out, adverse events, and psychological impact from utilising a WPAM. Our hypothesis was WPAM with exercise prescription or advice would improve CRF and step count, intensity of exercise, quality of life and, decrease sedentary time.

## Methods

A narrative synthesis, and meta-analysis, was performed in line with the protocol registered with PROSPERO, an international database of prospectively registered systematic reviews in health and social care (Registration Number: CRD42019106591) [50]. In January 2019, a systematic search of RCTs was completed by two authors (AH and MH) who followed the methodology proposed in the the Preferred Reporting Items for Systematic Reviews and Meta-Analysis (PRISMA) guidelines [51].

### Study selection

#### Inclusion criteria

This systematic review included RCTs, which were full-length research articles published in peer-reviewed academic journals. No limits were set on language, date of publication or gender. The RCTs must have compared standard care or an attention control group to the use of a WPAM during the maintenance phase (Phase 3) of CR. We define a WPAM to be a small, wearable device with accelerometer and/or pedometer capabilities. This may include pedometers, watches and smartphones (if the accelerometer function was used). To be eligible for inclusion, studies required at least 4 weeks follow-up after outpatient (Phase 2) CR. Standard care groups could include advice on PA and/or phone calls to encourage PA, however, not receive unblinded PA self-monitoring devices. The WPAM required data to be visible to the subjects in the intervention groups. Eligible studies included participants with a diagnosis of myocardial infarction, acute coronary syndrome; or who have undergone percutaneous coronary intervention, coronary artery disease; or a history of cardiac surgery (coronary artery bypass graft, valvular repair or replacement). Participants were required to be older than 20 years and must have completed Phase 2 of CR. Studies were required to have reported at least one outcome measure evaluating PA or CRF (e.g. change in peak oxygen uptake [VO_2_ peak] or change in steps per day). These outcome measures were used in the meta-analysis.

#### Exclusion criteria

Abstracts, poster presentations, conference presentations, unpublished books and letters to the editor or book chapters were excluded. Studies that used WPAM solely as an outcome measure, rather than an intervention, and which did not require participants to wear the devices throughout the entire study period, were excluded. In addition, studies that did not allow the participants to view the device data throughout the intervention period were also excluded.

### Literature search

Databases systematically searched included CINAHL, Cochrane Library, Embase, Medline/Ovid, Scopus, SPORTDiscus and Web of Science. A unique search strategy was identified, for each of the databases using the assistance of a university librarian and is available in the supplementary material. Reference lists of eligible articles and conference abstracts were also searched.

### Study selection

Two authors (AH and MH) independently conducted a systematic search to identify relevant titles and abstracts from the databases. Search results were entered into a reference management tool (Endnote v 9) and duplicates from different databases were removed. Both authors screened titles/abstracts for eligibility before viewing full text. In addition, reference lists of eligible studies were screened for further eligible studies. The primary author attempted to source full length text for eligible conference abstracts. The two reviewers compared studies for inclusion and exclusion. A third author (WH) was used to resolve discrepancies in decision making. The selection process was recorded into a PRISMA [51] diagram.

### Data extraction

For each RCT that met the inclusion criteria, the primary author (AH) completed the data extraction, which included author, year of publication, country of trial origin, number of participants, participant characteristics (gender, age and diagnosis), percentage of participants that completed the RCT, reasons for drop-out and adverse events. Furthermore, trial characteristics (type of wearable, timing of recruitment, length of trial and a description of the intervention) were also extracted. Finally, fitness and PA measures, specifically CRF and step count changes were collated.

This data entry was subsequently checked by a second author (MH). Discrepancies were resolved by a third author (WH). Authors of included studies were contacted if the paper stated relevant outcome measures were obtained, but not reported. Two authors [52, 53] were contacted and both provided additional information.

### Study quality

Methodological study quality was assessed and rated using the Physiotherapy Evidence Database Scale (PEDro Scale) which has been demonstrated to be a reliable and valid tool [54–56]. It identifies studies that are internally valid and was developed based on the Delphi list published by Verhagen et al. [57].

The PEDro-Scale ascertains the quality of reporting of studies. For this review, we allocated points if subjects were randomly allocated and concealed; participants had comparable baseline measures; subjects, therapists and assessors were blinded; more than 85% of starting subjects completed outcome measure assessment; participants received the allocated treatment; analysis included intention to treat; and if there was evidence of statistical comparison and variability of measures. Blinding of subjects or therapists was not possible because participants were required to wear a visible WPAM and therapists were required to discuss results of WPAM data with participants. Reporting of eligibility criteria is assessed for external validity; however, this is not included in the final score as per PEDro Scale marking requirements [55]. Therefore, removal of these criteria from the final scoring left a maximum possible score of 8. Two authors (AH and MH) independently used the PEDro scale’s criteria checklist to produce a score (between 0 and 8) to rate each studies’ quality. The same authors compared scores and discussed differences of opinion. Studies were deemed to be of good quality if the trial received a score of > 61% of available points (≥5/8). Fair-quality studies received 45.4–61% of available points (4/8). Studies which received < 45.4% of available points (< 4/8) were deemed of poor quality, as described by Kennelly et al. [58].

### Statistical analysis and synthesis

Review Manager (Version 5.3; The Nordic Cochrane Centre, Copenhagen) was used to perform a meta-analysis to investigate the effect wearing a WPAM with exercise prescription or advice had on CRF (change in VO_2_peak) and change in daily step count. Effect sizes for continuous variables were calculated as either mean difference or standardised mean differences (SMD), otherwise known as Cohens D effect size [59]. Standardised mean difference was used in cases where different methods across studies were used to assess CRF (treadmill test vs cycle ergometer) and because different types of WPAM were used across trials. The effect size was calculated as the difference in outcome measure reported from baseline to the end of the trial. Standardised mean difference (SMD) was used to quantify the effect size in place of mean differences (steps per day) due to standard deviations being too wide for visual representation. Sub-groups were used to represent the overall influence of effect; where SMD > 0.8 represented a large effect, 0.5–0.79 represented a moderate effect, and 0.2–0.49 a weak effect. This has been used in previous research [59].

Where standard deviation of the change was not published, we estimated it using the *p*-value between groups, then within groups, as recommended by the *Cochrane Handbook for Systematic Reviews of Interventions* [60]. Random effects with standardised means model was implemented due to the variability of duration, delivery and assessment across studies. Raw data was received from ter Hoeve et al. [52] as the actual step count in their publication was not reported. We therefore derived the steps by entering the raw data into statistical software package (IBM SPSS Statistics, version 25) and performed a paired t-test.

A forest plot was completed on CRF changes (VO_2_peak) and step count per day. These were the only outcome measures found in three or more studies. Heterogeneity, using I^2^ was considered significant at *p* < 0.1. If I^2^ was 0–30%, it was considered minimal, 31–50%, moderate, 51–90% substantial and considerable if > 90% [61].

Finally, due to the small number of studies that were included in the meta-analysis, we also performed a qualitative best evidence synthesis. This was considered as inferior evidence to the quantitative analyses’ method of meta-analyses. This method was based on previous research [62] which provided recommendations on how to conduct a qualitative analysis using five levels of evidence from strong to no evidence. A best evidence synthesis approach has widely been used within systematic reviews where quantitative approaches are not possible [63–66]. From this, we adapted the criteria due to the small number of studies in our review as below:

1. Strong Evidence: significant findings provided by two or more studies with high quality and by generally consistent findings in all studies (more than 75% of the studies reported consistent findings).
2. Moderate Evidence: significant findings provided by one study with high quality and/or two or more studies with low quality, and by generally consistent findings in all studies (more than 60% of the studies reported consistent findings).
3. Limited Evidence: significant findings provided by only one study with low quality.
4. Conflicting Evidence: inconsistent findings in multiple studies (less than 60% of studies reported consistent findings).
5. No Evidence: when no studies could be found

## Results

**Fig. 1:**
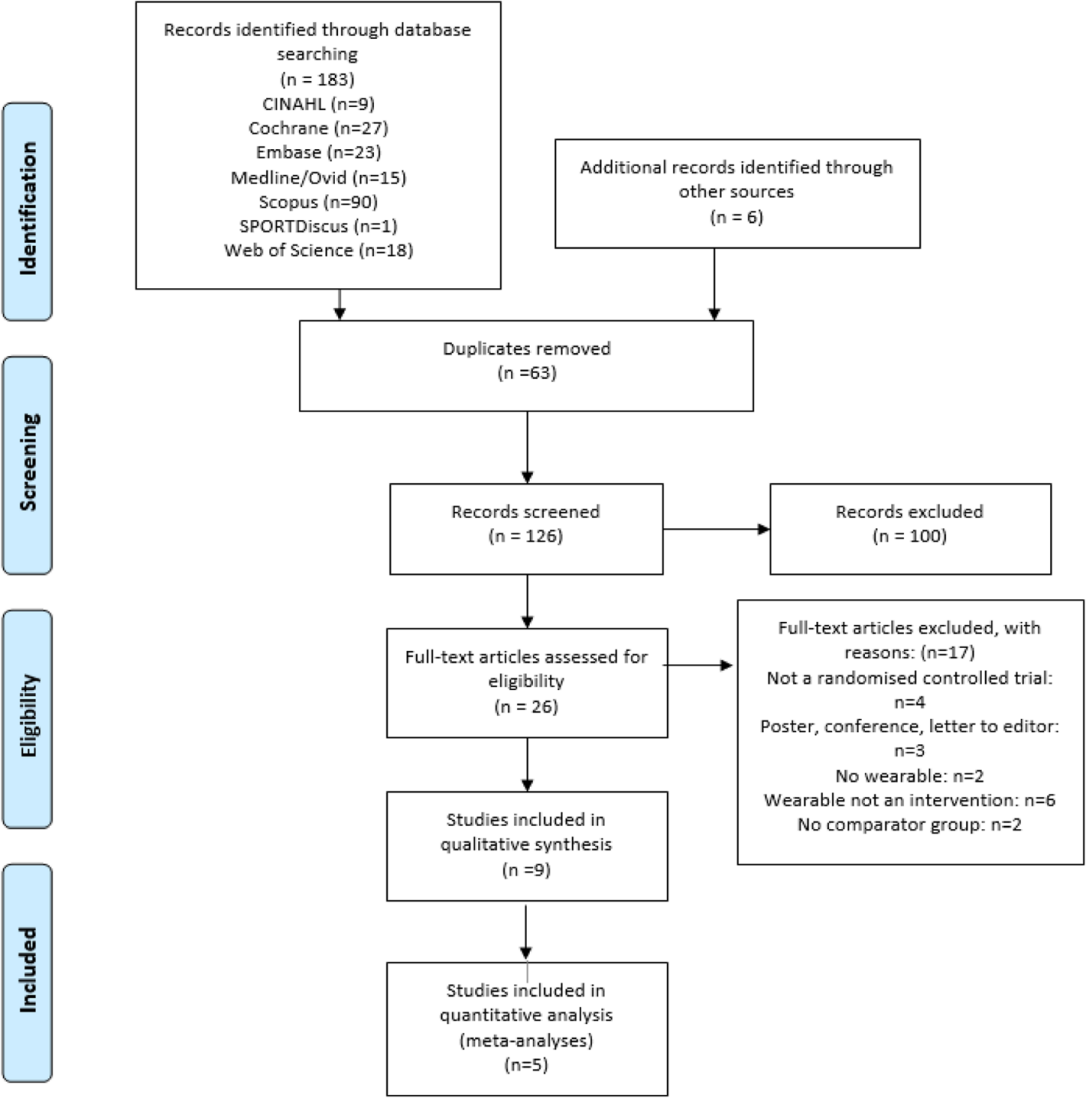
PRISMA diagram of literature search strategies

Initially, the search strategy resulted in 183 articles. This was reduced to 126 articles after duplicates were removed. The titles and abstracts were screened, and 100 studies were excluded due to not meeting eligibility criteria. Of the 26 articles that were screened, nine were identified as meeting the inclusion criteria for the systematic review (Fig. 1).

### Study quality

The PEDro-Scale was used to gauge the quality of individual trials. Nine studies were scored by two authors (AH and MH) independently and discrepancies were discussed and agreed. Of the nine studies, none were of poor quality, two were of fair quality (2/9) and seven were of good quality (7/9) (Table 1).

**Table 1:** Quality Analysis using PEDro-Scale (Cross indicates study did not meet this criteria)

	Eligibility Criteria Specified (Not included in final score)	Randomly Allocated	Allocation Concealed	Similar Baseline Measure-ments	Blinding of Assessors	Less than 15% dropout both groups	Intention to Treat	Statistical Comparisons	Variability Measures	Score
Avila et al. [67]	✓	✓	X	✓	X	✓	✓	✓	✓	6
Butler et al. [68]	✓	✓	X	✓	X	X	✓	✓	✓	5
Cupples et al. [69]	✓	X	X	✓	X	✓	X	✓	✓	4
Duscha et al. [70]	✓	✓	X	✓	X	X	X	✓	✓	4
Guiraud et al. [71]	✓	✓	X	✓	X	✓	✓	✓	✓	6
Houle et al. [72]	✓	✓	X	✓	X	✓	X	✓	✓	5
Skobel et al. [73]	✓	✓	X	✓	✓	X	X	✓	✓	5
ter Hoeve et al. [52]	✓	✓	✓	✓	X	X	✓	✓	✓	6
Varnfield et al. [53]	✓	✓	✓	✓	X	X	X	✓	✓	5

### Study characteristics

All eligible studies were published in English and were included in the narrative analysis [52 53,67–73]. Five studies (56%) were from Europe [52, 67, 69, 71, 73] including Belgium (*n* = 1) [67], Ireland (n = 1) [69], France (n = 1) [71], Netherlands (n = 1) [52] and a multicenter study across Germany, Spain and Britain [73] (n = 1). Two studies (22%) were based in Australia [53, 68], one was from the United States of America [70], and one study from Canada [72] The studies were published between 2009 and 2018.

The total number of participants across all studies was 1,352. Of the 870 participants that were analysed,192 (22.1%) were female and 678 (77.9%) were male. The lowest percentage (4%) of a specific gender in the control and intervention groups across studies was females in a single control group [69]. Mean ages of participants ranged from 42 to 73.7 years. In five studies [52, 53, 69, 72, 73] (56%), the mean age for the control group was < 60 years (range 56.2+/− 10.1 to 59.1+/− 8) and four studies [67, 68, 70, 71] reported mean ages > 60 years (range 61.7+/7.7 to 66.5+/− 7.2). In contrast, six studies [52, 53, 67, 70–72] (67%) reported the mean age for the intervention group as < 60 years (range 54.5+/− 12.6 to 59.9+/− 8.1) and three studies [68, 69, 73] reported the mean ages as ≥60 years (range 60–63+/− 10.4). Seven studies [52, 53, 67, 68, 70–72] (78%) reported younger mean ages for the intervention group compared to the control group, however this was not significantly different. Common presentations of participants reported by studies included myocardial infarction (*n* = 7), coronary artery bypass graft surgery (*n* = 6), percutaneous coronary intervention (n = 7), acute coronary syndrome (*n* = 2), and coronary artery disease (n = 2). Four studies [67, 69–71] (44%) had durations between 1.5 and 3 months in length, three studies [53, 68, 73] (33%) were 6 months duration and two studies [71, 72] (22%) were longer than 6 months duration. Individual trial breakdowns for patient characteristics can be seen in Table 2.

**Table 2:** Study and Participant Characteristics

Study	Country of Origin	No. Participants	Gender (f/m)	Age (years±SD)	Diagnosis	Study Duration (months)	% of participants completed study
Avila et al. [67]	Belgium	30 c 60 i 84^a^	3/27 c 4/26 i	61.7 ± 7.7 c 58.6 ± 13 i	CAD, MI, CABG, PCI	3	86.67 c 93.33 i
Butler et al. [68]	Australia	60 c, 62 i 6/52: 50 c, 48 i; 98^a^ 6/12: 46 c, 44 I; 90^a^	10/45 c 17/38 i	64.5 ± 11.2 c 63 ± 10.4 i	MI, CABG, PCI, ACS	6	6/52: 90.9 c; 87.3 i 6/12: 83.64 c; 80 i
Cupples et al. [69]	Northern Ireland	26 c 19 i	1/25 c 3/16 i	59.2 ± 8.9 c 61.6 ± 11.3 i	Not published	1.5	96 c 90 i
Duscha et al. [70]	America	11 c 21 i; 25^a^	3/6 c 3/13 i	66.5 ± 7.2 c 59.9 ± 8.1 i	MI with PCI or CABG, PCI, CABG, VR	3	81.8 c 76.2 i
Guiraud et al. [71]	France	10 c 19 i	3/7 c 2/17 i	62.9 ± 10.7 c 54.5 ± 12.6 i	CAD, CABG, PCI, HF	2	100 c 100 i
Houle et al. [72]	Canada	33 c 32 i	8/25 c 6/26 i	59 ± 9 c 58 ± 8 i	MI, CABG, PCI, UA	12	Data not published
Skobel et al. [73]	Germany, Spain, Britain	63 c 55 i 54^a^:42 c,12 i	8/55 c 5/ 50 i	58 c* 60 i*	MI, PCI	6	66.7 c, 21.8 i
ter Hoeve et al. [52]	Netherlands	163 c 161 i	32/131 c 32/129 i	59.1 ± 8 c 58.8 ± 9 i	MI, CABG, PCI, ACS	18	3/12: 78 c, 80.1 i 12/12: 75 c,75 i 18/12: 74.7 c,74.8 i
Varnfield et al. [53]	Australia	41 c 53 i 6/52:28 c 48 i; 76^a^ 6/12: 26 c, 46 i; 72^a^	7/34 c 5/48 i	56.2 ± 10.1 c 54.9 ± 9.6 i	MY	6	6/52:46.7 c, 80 i 6/12:43.3 c,76.7 i

*Abbreviations*: *f* female, *m* male, *SD* standard deviation, *t* total, *c* control, *i* intervention, ^a^ analysed, *CAD* coronary artery disease, *MI* myocardial infarction, *CABG* coronary artery bypass graft surgery, *PCI* percutaneous coronary intervention, *ACS* acute coronary syndrome, *VR* valve repair, *HF* heart failure, *UA* unstable angina, *wks* weeks, *m* months,^a^: *SD not published

All studies utilised a WPAM for the intervention group. The devices utilised included Yamax Digiwalker Pedometer [52, 68, 69, 72], Garmin Forerunner [67], Fitbit Charge [70], My Wellness Key Accelerometer [71], Gex vital signs sensor [73], Nokia Smartphone with pre-installed applications [53], and a Sensewear Mini Armband [67]. The timing of recruitment for study participants ranged from 6 weeks to 18 months post cardiac event. Participants completed a supervised phase 2 CR program prior to participation in all studies, except one [53]. The one exception commenced the intervention period at the onset of phase 2 CR and continued into phase 3 with final outcomes measured at 6 months [53].

The duration of interventions varied across studies. In seven studies [52, 53, 67, 68, 70–72] (78%), the control group received a pamphlet and/or face-to-face sessions on PA and lifestyle factors. One study [69] included ongoing weekly facilitator support for the attention control group, and another had participants in the control group report daily PA in a paper diary [73]. Seven of the studies (78%) included goal setting in the interventions. This was performed by phone calls, emails, text messages or a web-based interface [53, 67–71, 73]. One study received a socio-cognitive intervention led by a clinical nurse specialist [72]. All studies encouraged self-management using the WPAM to track their PA. Individual trial characteristics can be seen in Table 3. Five (56%) of the studies exercise interventions were based at home [69–73], three (33%) used both home-based and centre-based locations [52, 53, 67] and one study did not report on the location of exercise [68]. There was a large variety of different recommended parameters for individual exercise sessions. Thirty minutes of daily moderate intensity activity was recommended to the control group participants in two studies [68, 69] (22%) whilst two others (22%) reported general advice to stay active [70, 71]. Exercise parameters for home-based exercise were all unique and exercise prescription varied in the amount of specific instruction given to intervention participants. One study did not specify any exercise prescription [72] and another three (33%) provided general advice only; to exercise at moderate intensity for most days of the week [53, 67, 71]. Two studies (22%) recommended increasing steps per day [69, 70], two (22%) prescribed a specific heart rate range and duration [67, 68]. Two further studies (22%) incorporated additional exercise modes, other than walking (resistance training [73] and gymnastics [52]). Only one study [73] gave participants a detailed prescription on how to progress the exercise, however this was only for the centre-based participants. Individual trial breakdowns for study characteristics can be seen in Table 3.

**Table 3:** Study Parameters

Study	Type of wearable	Timing of recruitment	Intervention Description			
			control	intervention	parameters for individual sessions	
					control	intervention
Avila et al. [67]	Garmin Forerunner 210 Sensewear mini armband	3 months post ambulatory CR	advised to remain physically active	home-based exercise with telemonitoring guidance weekly emails or phone calls centre-based	data not published	150 mins of activity/week 6–7 days /week moderate intensity exercise (70–80% Heart rate reserve)
Butler et al. [68]	Pedometer Yamax Digiwalker 700B	following attendance of group CR	given 2 generic PA brochures	6-week self-monitored activity with pedometer, daily step calendar, generic PA brochure approximately 15-min-long phone call after 1,3,12,18 weeks 2 behavioural counselling and goal setting sessions week 1 and 3	30 mins of moderate intensity activity on all or most days of the week	data not published
Cupples et al. [69]	Pedometer Yamax CW-701	following completion of supervised CR	ongoing weekly facilitator support but no feedback on step counts	worked with a clinical facilitator pedometer set daily step count goals with weekly reviews record daily steps in a diary home-based	30 min of moderate intensity activity daily	gradual increase of 10% of steps aiming for 10, 000 steps/day
uscha et al. [70]	Fitbit Charge	2 weeks prior to discharge from group CR	patients wore Fitbit during last 2 weeks of group CR usual care as advised by physician Fitbit worn for last 2 weeks of study	patients wore Fitbit during last 2 weeks of group CR plus following 12 weeks exercise prescription of step counts weekly health coaching (1–2 times/week for 30–60 min) text messages and educational material Vida Health app home-based	advice given by individual physicians specifics not published	weeks 1–4 increase PA by 2,500 steps above baseline weeks 5–8 increase a further 1,250 steps weeks 9–12 increase a further 1,250 steps
Guiraud et al. [71]	My Wellness Key Accelero-meter	2 months or 1 year after discharge from group CR	wore accelero-meter in last week only advice on importance of adhering to exercise prescription given	accelerometer worn throughout telephone support given every 15 days identifying barriers and strategies home-based	no contact given	moderate intensity PA
Houle et al. [72]	Yamax Digiwalker NL-2000 – blinded Yamax Digiwalker SW − 200	within 4 weeks of discharge from hospital	usual advice by nurse or physician no restrictions to go to centre-based CR	pedometer PA diary Socio-cognitive intervention led by clinical nurse specialist home-based	usual advice- specifics not published	given pedometer-based program
Skobel et al. [73]	Gex sensor of vital signs and smartphone	during group CR	Report PA in paper diary	Guided exercise system (Gex) individual performances monitored and exercise prescription reviewed web based tool, patient station and portable station home-based	specifics not reported	endurance training plus resistance training (both isometric and isotonic exercises using a rubber band) Week 1–3; 2 x wk., 3 × 10 mins, Borg 11 Week 4–6; 2 x wk., 3 × 10 mins, Borg 12–13 Week 7–9; 2 x wk., 3x15mins, Borg 12–13 Week 10–12; 3 x wk., 3x15mins, Borg 12–13 Week 12+; 3+ x week, 3 × 20 mins, Borg 12–13
ter Hoeve et al. [52]	Yamax Digiwalker SW-200 Tri-axial accelerometer over 8-day period	during group CR	standard CR for 3 months no after care general information of benefits of PA	Standard CR for 3 months + 3 face to face group PA counselling sessions and pedometers. Booklet with goal setting barrier identification and relapse strategies. Education about sedentary time given home-based and centre-based	2 x week 75 mins gymnastics, walking sports for 3 months followed by no after care	2 x week75 mins gymnastics, walking sports for 3 months 9 months after care program: 3 face to face sessions: 1-h exercise program and 1-h behavioural counselling program
Varnfield et al. [53]	CAP-CR via Nokia smartphone pre-installed with step counter and health diary with accelero-meter	patients eligible for a CR referral average day post cardiac event: control: 68 days CAP-CR: 53 days	centre based CR for 6 weeks encouraged to maintain lifestyle changes achieved during CR	CAP-CR App home-based and centre-based	2 x week exercise for 6 weeks circuit based exercise light to moderate intensity followed by self-management	weekly telephone consultation: 15 min each for 6 weeks 30 mins exercise most days of the week moderate intensity walking followed by self-management

*Abbreviations*: *CR* cardiac rehabilitation, *PA* physical activity, *CAP-CR* care assessment platform

### Reasons for drop out

Completion rates amongst trial groups ranged from 22% [73] to 100% [71]. The collective mean drop-out rate percentage was slightly lower for the intervention groups compared with the control groups (22% versus 23% respectively). For studies of less than or equal to 3 months (4/9), the mean dropout rate for the control groups was 9 and 10% for the intervention group. For studies greater than 6 months (5/9), the mean dropout rate for the control groups was 31 and 34% for the intervention group. Common reasons participants dropped out of studies included loss of interest/withdrew [52, 67, 68, 73], family commitment [68], work commitment [53, 68], medical reasons [52, 53, 68, 69, 73], lack of time [53, 73], technical issues [53, 73], and lack of motivation [52, 53, 70, 73]. Individual trial breakdowns for reasons for drop out can be seen in Table 4.

**Table 4:** Reasons for Drop Out and Adverse Events

Study	Reasons for Drop Out (n)			Adverse Events
	control	intervention	unclassified/other	
Avila et al. [67]	loss of interest (2) new cardiac intervention (2)	loss of interest (2)		nil events occurred
	6- week follow up: unrelated medical reasons (3) work (1) withdrew consent (1) excluded (5)	6- week follow up: unrelated medical reasons (4) work (1) withdrew consent (1) excluded (7)		data not published
Butler et al. [68]	6- month follow up: unrelated medical reasons (3) deceased (1)	6- month follow up: unable to be contacted (2) family needs (1) work (1)		
Cupples et al. [69]	influenza (1)	anaemia (1) depression (1)		ankle injury knee injury back pain shortness of breath (no events prevented completion of study)
Duscha et al. [70]	reason not published (2)	reason not published (3) unusable data: failed to give a good effort on CPX; ICD reset (2)	lost to follow up (2)	randomised group not published knee injury from falling on ice rare blood disease diagnosis severe fishing hook wound
Guiraud et al. [71]	nil	nil	nil	nil events occurred
Houle et al. [72]	data not published	data not published	data not published	data not published
Skobel et al. [73]	withdrew (18) cancelled follow up (3)	withdrew (15) poor compliance (17) lack of time, internet issues, demotivation (21) chronic infection (1) back pain (1)	technical problems (21)	control; new onset atrial fibrillation (1) new angina at rest (1) pseudo aneurysm of femoral artery after PCI (1) intervention: none related to exercise patients required angiography (not related to training) (2) chest pain requiring CABG before exercise (2)
ter Hoeve et al. [52]	lost to follow up (62) prematurely quit (52) declined further participation: poor motivation (5) unknown (4) medical complications (1)	pedometer: lost to follow up (57) prematurely quit (43) declined further participation: poor motivation (5) unknown (8) medical complications (1)		data not published
Varnfield et al. [53]	logistical: time16% location 7% transport 24% competing life demands: work 10% stress 4% change in circumstances: deterioration of health unrelated to CR 14% lack of motivation 4%	change in circumstances deterioration of health unrelated to CR 9% difficulty using IT tools 7%		data not published

*Abbreviations*: *CPx* cardiopulmonary exercise test, *ICD* implantable cardioverter-defibrillator, *PCI* percutaneous coronary intervention, *CABG* coronary artery bypass graft surgery, *CR* cardiac rehabilitation, *IT* information technology

### Adverse events

Five studies (56%) reported adverse events during the trial period [67, 69–71, 73]. No adverse events related to exercise occurred in two of these studies [67, 71]. Adverse events, which were non-cardiac related (ankle [69], knee [69, 70] and back injuries [69], shortness of breath [69], rare blood disease [70] and fishing hook wound [70]), were reported in two studies [69, 70]. One study [73] reported adverse cardiac events, of which there were seven incidents (new onset atrial fibrillation, new onset angina at rest and femoral artery aneurysm post percutaneous coronary intervention), however, none were deemed to be related to exercise. Individual trial breakdowns for adverse events reported can be seen in Table 4.

### Outcome measures

Outcome measures used in the studies were varied. CRF was assessed by five studies [53, 67, 68, 70, 73] (56%). Step count was measured by five studies [52, 67, 69, 70, 72] (56%) over a one [52, 69, 72] or 2 week [70] period, using pedometers [69, 72] or accelerometers [67, 70, 71]. Avila et al. [67] did not report the time period that step counts were measured. Exercise duration was reported by three studies [67–69] (33%), exercise intensity was reported by four studies [52, 67, 70, 71] (44%), and two studies reported on sedentary time [52, 67]. Individual trial outcome measures can be seen in Table 5.

**Table 5:** Physical Activity Outcome Measures

Study	Steps/Day		VO_2_ peak (mean ± SD)		Physical Activity Duration (mean mins ± SD)		METS at AT	
Avila et al. [67]	Pre	Post	Pre	Post	Pre	Post		
	6419 (2227–13181) c^b^	6408 (296–12041) c^b^	26.6 ± 4.9 c	26.4 ± 5.4 c	114 ± (30-311) c ^e^	114 ± (6-382) c ^e^		
	7896 (2018–12554) i^b^	6469 (473–12554) i^b^	26.7 ± 6.6 i	27.8 ± 6.8 i *p* = 0.03	145 ± (34–299) i^e^	141 ± (51–259) i^e^		
Cupples et al. [69]	7869 ± 4209 c ^a^	42 ± 2,624 c^ch^						
	6123 ± 3151 i ^a^	2742 ± 3164 i ^ch^ *p* = .004						
Duscha et al. [70]	7411 ± 2811 c ^a^	7243 ± 3209 c ^a^	20.7 ± 5.6 c	19.1 ± 5.5 c				
	9003 ± 2694 i ^a^	9414 ± 3051 i ^a^	21 ± 5.7 c	21.7 ± 5.6 c				
Houle et al. [72]	41 c^d^	55 c^d^						
	31 i^d^	83 i^d^ *p* = .042						
ter Hoeve et al. [52]		514 ± 115 c^ch^						
		1504 ± 1835 i ^ch^						
Skobel et al. [73]			12.8^a^ c	19.5 ± 4.8				
			13.8^a^ i	21.9 ± 8.3 *p* = .005				
Butler et al. [68]					367 ± 268 c^f^	355 ± 271 c^f^	3.6 ± 0.8c	3.9 ± 1.3c
					343 ± 275 i^f^	455 ± 361 i^f^ *p* = .025	3.5 ± 0.7 i	3.9 ± 1.1 i

*Abbreviations*: *c* control group, *i* intervention group, *SD* standard deviation,^a^_:_ mean ± SD,^b^: mean (range),^ch^: resulting change mean ± SD,^d^: % of participants achieving > 7500 steps/day, %: percentage, VO_2_peak: maximal oxygen uptake, *METS* metabolic equivalents, *AT* anaerobic threshold, *p*: *p* value,^e^:> 3 METS; mins/day± (range),^f^: mins/week; mean ± SD; Active Australia Survey

**Fig. 2:**
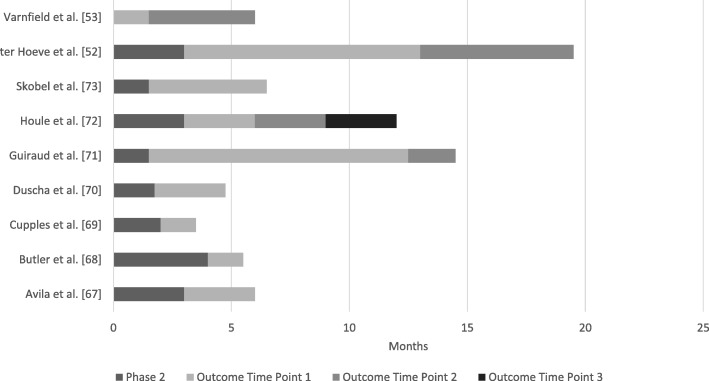
Duration of Study

Timepoints for outcome measure acquisition can be seen in Fig. 2. The figure shows the wide range of study lengths (12 weeks to 18 months) and timing of main outcome measures. Two studies [69, 70] (22%) were less than 6 months’ duration, four studies [53, 67, 68, 73] (44%) were between five- and seven-months’ duration, and three studies [52, 72, 72] (33%) ran for 12 months or more. The longest duration was 18 months [52]. Duscha et al. [70] did not report the length of time of Phase 2 CR, only the number of sessions, and Guiraud et al. [71] used two groups of participants that had either 2 months or 12 months of no intervention between the completion of their Phase 2 CR and the onset of the Phase 3 CR.

### Cardiorespiratory fitness/exercise capacity

Five studies [53, 67, 68, 70, 73] (56%) measured CRF/exercise capacity changes. The outcome measure varied across studies. Three studies [67, 70, 73] (33%) measured VO_2_peak. Two of these, used a cycle ergometer with expired gas analysis [67, 73] and one used a maximal treadmill test with expired gas analysis [70]. Another study measured anaerobic threshold using a cycle ergometer with gas analysis [68] and one study [53] utilised a six-minute walk test (6MWT).

**Fig. 3:**
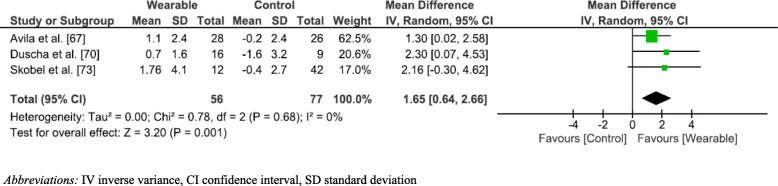
Forest Plot aerobic capacity

**Fig. 4:**
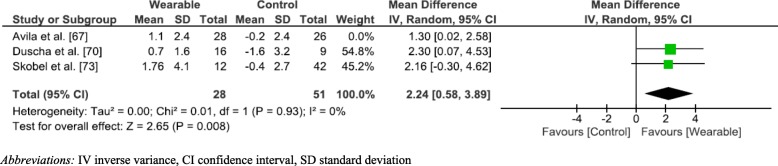
Sensitivity analysis Vo2peak

Figure 3 depicts the meta-analysis and forest plot results performed for VO_2_peak changes. The meta-anlaysis identified three studies [67, 70, 73] that had assessed change in VO_2_peak. All three studies showed WPAM with exercise prescription or advice significantly improved VO_2_peak as compared to not utilising a WPAM; (MD 1.65 mL/kg/min; 95% CI [0.64–2.66]; *p* = 0.001; I^2^ = 0%). A sensitivity analysis was performed by removing Avila et al. [67]. This resulted in a larger mean difference (1.65 [0.64–2.66] versus 2.24 [0.58–3.89]). The heterogeneity remained at I^2^ = 0%. (Fig. 4). A sensitivity analysis that removed the weighting of Avila et al. [67] was employed due to the heterogeneity of the results of daily step count compared to the three other studies included in the meta-analysis. The VO_2_ peak results extracted from Avila et al. [67] were, however, significantly more homogenous. Avila et al. [67] utilised a heart rate monitor (Garmin Forerunner) to guide the participant’s exercise sessions. Therefore, participants of this study may not have engaged solely in walking or running during the intervention period, but rather may have chosen numerous other forms of exercise such as cycling or gymnastics.

A qualitative analysis of the effect WPAM with exercise prescription or advice had on CRF/physical capacity was undertaken. The analysis showed a moderate level of evidence for WPAM improving physical capacity to a greater extent than no WPAM (Table 6).

**Table 6:** Qualitative Analysis of Physical Capacity Outcome Measures

	Study Quality	Outcome Measure	Effect	Best Evidence Synthesis
Avila et al. [67]	Good	VO _2_ peak	+	Moderate ^a^
Butler et al. [68]	Good	METs at AT	=	
Duscha et al. [70]	Fair	VO _2_ peak	+	
Skobel et al. [73]	Good	VO _2_ peak	+	
Varnfield et al. [53]	Good	6MWT	=	

+, significant difference favouring WPAM, −, significant difference favouring control, =, no significant difference between groups. ^a^Moderate Evidence: significant findings provided by one study with high quality and/or two or more studies with low quality, and by generally consistent findings in all studies (more than 60% of the studies reported consistent findings)

*Abbreviations*: *VO*_*2*_*peak* peak aerobic capacity, *METs* metabolic equivalents, *AT* anaerobic threshold, *6MWT* six-minute walk test

### Six-minute walk test

Varnfield et al. [53] completed a 6MWT test to determine the impact of their Care Assessment Platform CR intervention on exercise capacity at 6 weeks and 6 months. They found both groups significantly increased distance from baseline to 6 weeks and 6 months, however there was no significant difference between groups. (6 weeks: control 537 ± 86 m to 584 ± 99 m; *p* = 0.001 vs intervention 510 ± 77 m to 570 ± 80; *p* < 0.001); (6 months: control 537 ± 86 m to 601 ± 95; *p* < 0.05 vs intervention 510 ± 77 m to 571 ± 88; *p* < 0.05). Adjusted mean difference at 6 weeks was not found to be significant *p* = 0.4.

### Pedometer step count

**Fig. 5:**
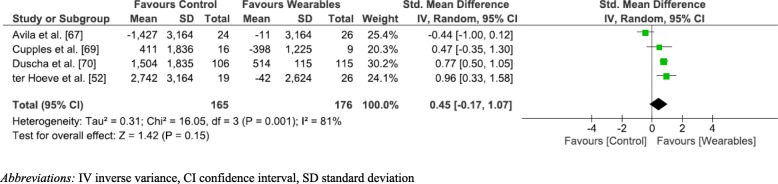
Step count

Five studies [52, 67, 69, 70, 72] (56%) reported on the number of steps completed by participants. Fig. 5 depicts the meta-analysis and forest plot results performed for step count change pre and post intervention. Three studies [52, 69, 70] (75%) showed improved step counts when using a WPAM versus not utilising one, however the overall effect was not significant. (SMD 0.45; 95% CI [− 0.17–1.07]; *p* = 0.15; I^2^ = 82%). The SMD of 0.45 equates to a medium effect size.

**Fig. 6:**
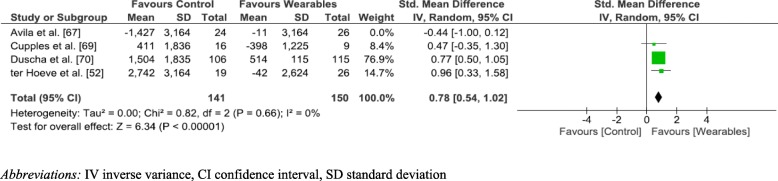
Sensitivity Analysis step count

A sensitivity analysis was performed by removing Avila et al. [67]. This resulted in a significant difference in step count, favouring WPAM with exercise prescription or advice (SMD 0.78; 95% CI [0.54–1.02]; *p* < 0.001). The increased SMD of 0.78 also equated to a moderate effect size. Removing Avila et al. [67] also reduced the heterogeneity to 0% (Fig. 6).

Houle et al. [72] also reported on steps of participants, however published percentage of participants reaching > 7,500 steps per day and therefore could not be included in the meta-analysis. They did find a significant difference between groups. The intervention group significantly increased the percentage of patients achieving > 7,500 steps per day more than the control group at 6,9 and 12 months (75% vs 41%;68% vs 36%;83% vs 55%,respectively; *p* < 0.05).

A qualitative analysis of the effect WPAM with exercise prescription or advice had on the amount of PA performed by participants was undertaken. The analysis showed a moderate level of evidence for WPAM improving PA to a greater extent than no WPAM (Table 7).

**Table 7:** Qualitative Analysis of Physical Activity Outcome Measures

	Study Quality	Outcome Measure	Effect	Best Evidence Synthesis
Avila et al. [67]	Good	Steps/day and PA Duration	=	Moderate ^a^
Butler et al. [68]	Good	PA Duration	+	
Cupples et al. [69]	Fair	Steps/day	+	
Shower and a. [70]	Fair	Steps/day	=	
Guiraud et al. [71]	Good	Total Active Energy Expenditure	+	
Houle et al. [72]	Good	% of participants over 7,500 steps/day	+	
ter Hoeve et al. [52]	Good	Steps/day	+	

**+,** significant difference favouring WPAM, −, significant difference favouring control, =, no significant difference between groups. ^a^Moderate Evidence: significant findings provided by one study with high quality and/or two or more studies with low quality, and by generally consistent findings in all studies (more than 60% of the studies reported consistent findings)

*Abbreviations*: *PA* physical activity, % percentage

### Intensity/Accelerometry data

Four studies [52, 67, 70, 71] (44%) reported on intensity of PA. One study [67] did not record how long the intensity was measured for. Another study [71] recorded intensity data throughout the intervention period in the wearable group and for 1 week in the control group. A third study [52] collected intensity data over 8 days, and the final study [70] recorded intensity data for 2 weeks at the beginning and 2 weeks at the end of the intervention period.

One study found no significant differences in the intensity of exercise performed by participants in either the intervention or control group [67]. The three remaining studies [52, 70, 71] reported different findings. One study [70] reported the intervention group significantly increased the time spent in moderate-high intensity activity compared to the control group (intervention; 3 ± 15 mins/day increase versus control; − 7 ± 5 mins/day decrease; *p* < 0.05). In addition, the authors found the control group significantly decreased the time in moderate-low and moderate-high intensity. The change between the groups was significant in both categories (moderate-low; − 10 ± 1 2 mins/day *p* < 0.05; moderate-high − 7 ± 5 mins/day; *p* < 0.05). The second study [71] reported the duration of moderate intensity PA increased significantly at the 8 week compared to baseline in the intervention group only (70.1 ± 32.4 min/week to 137 ± 87.5 min/week);(*p* < 0.0004). The final study [52] showed no significant change in moderate to vigorous intensity PA between control and intervention groups (*p* = 0.529), however time in prolonged moderate-vigorous PA of the intervention group improved more at 3 months compared with the control group. (*p* = 0.054).

### Sedentary time

Two studies reported on changes to time spent sedentary [52, 67]. Avila et al. [67] found no significant differences between the control and home-based groups (control: 1100; range: 825–1355 min/day to 1062; range: 484–1402 versus intervention: 1039 range: 688–1260 to 1032 range:790–1455 min/day). In addition, ter Hoeve et al. [52] also reported no change in sedentary behaviour time.

### Psychological measures/quality of life

A third of the studies (3/9) did not have outcome measures to investigate the effect of WPAM with exercise prescription or advice on quality of life (QoL) or psychological factors [52, 70, 71]. Each study that did assess psychological effects (6/9) used different tools, however the EQ. 5D and Kessler scales were used in several studies. Three [67, 69, 73] of the six studies [53, 67–69, 72, 73] that used psychological outcome measures found no significant differences in health related quality of life [67, 69], general health status (EQ. 5D) [69, 73], hospital anxiety and depression scale [73] or stage of behavioural change [69]. However, the remaining three studies [53, 68, 72] did report significantly improved overall quality of life [72], health related quality of life [53], general health status (EQ. 5D) [53] and decreased depression, anxiety and stress scale (DASS21) [53] and psychological distress scale (Kessler 6 [68] and Kessler 10 [53]) scores.

Specifically, Butler et al. [68] reported the intervention group had significantly greater improvement in behavioural (*p* = 0.039); and cognitive strategies (*p* = 0.024) compared to the control group at 6 weeks, however, at 6 months only the cognitive strategies remained significantly greater when adjustments were made for baseline differences (*p* = 0.001).

At 6 weeks, Varnfield et al. [53] reported significant improvements in several components of the Kessler 10 for both groups, however, these were not significantly different between groups (psychological distress scale, DASS-anxiety). The EQ. 5D scores significantly improved in the intervention group compared with the control group (*p* < 0.001). At 6 months, the between group differences were not significant for Kessler 10 nor EQ. 5D.

Houle et al. [72] used the Quality of Life Index-cardiac version 111 and reported the health and functioning score (*p* = 0.048) and family score (*p* = 0.048) were statistically improved compared to control group at 6 weeks. They also found overall QoL (*p* = 0.048) and the health and functional score (*p* = 0.036) were significantly improved compared to the control group at 12 months.

## Discussion

The aim of this systematic review and meta-analysis was firstly to determine whether using a WPAM with exercise prescription or advice during the maintenance phase of CR was effective in maintaining or improving CRF and/or the amount of daily PA and sedentary time. Secondly, we aimed to collate the outcome measures used in the studies, reasons for drop out, adverse events, and QoL/psychological impact resulting from WPAM during the maintenance phase of CR. Our review of the literature identified that there are no other systematic reviews investigating the effect of WPAM on the above parameters within the cardiac population.

### Main findings

The main findings of the reviewers were that using a WPAM with exercise prescription or advice significantly improved CRF to a greater extent than having no device for people with cardiac disease who are exercising through to the maintenance phase of CR. The review also showed that WPAM did not result in any cardiac adverse events and may assist in improving step count and some components of psychological measures (cognitive and behavioural strategies, psychological distress, anxiety, overall QoL).

### Study quality

Overall, our results showed the quality of individual studies in our review was good. When scoring the methodology of the studies using the PEDro scale, only two studies [69, 70] were found to be of fair quality. In future studies, the addition of blinding assessors and incorporating intention to treat in data analysis, would assist in improving study quality.

### Study characteristics

Our results showed that research into the effectiveness of WPAM in the cardiac population, although limited, has been conducted primarily in the northern hemisphere (78%), with only two studies occurring south of the equator. Most participants across the included studies were male (78%), which represents male dominated enrolment seen in CR [74]. Future studies investigating whether the effects of WPAM and exercise prescription or advice differ depending on sex would be beneficial.

The participant diagnoses and cardiac interventions across the studies represented the main patient presentations seen at CR programs and therefore, were a good representation [5]. However, no studies investigated whether patients’ specific diagnosis influenced the outcomes from WPAM. This would be valuable for future studies as this could ascertain, for instance, whether patients who are re-perfused benefit more from utilising a WPAM than those on medical management.

There were many different WPAM used across studies. Therefore, the results need to be viewed with caution as none of the studies included in this review compared the effectiveness of different devices at increasing PA. A study by Cadmus-Bertram et al. [75] showed Fitbits to be more effective than pedometers at increasing exercise intensity of participants, therefore comparisons between devices would be useful.

Our review noted that the duration of less than half the studies was 3 months or less and the longest study duration was 18 months. Most studies, therefore, were too short to predict the effect of WPAM with exercise prescription or advice on mortality, hospital admission and long-term adherence to PA. Our results found greater dropout rates were seen in the studies lasting more than 6 months compared with those lasting three or fewer months. Longer duration studies are warranted to determine whether adherence to the usage of a WPAM decreases over time.

There were large variations across studies regarding exercise advice given to participants, recording practices of daily exercise, and additional input given to improve adherence. It is difficult to determine whether WPAM alone are responsible for the improvements shown and what contribution these confounding variables may have made to the results.

### Reasons for drop out

According to a review by Dishman et al. [76], 50% or more of participants drop out of exercise in clinical settings within 6 months. Apart from one study [73], the dropout rate for the studies in our review was found to be less (< 33%) than this. Our results also suggested that using a WPAM did not affect the dropout rates compared to using no device.

The review by Dishman et al. [76] reported that attitudes to exercise, self-perceptions, health beliefs, goals, and motivation were the main influencing factors to adherence. Our findings were similar as most participants reported lack of interest and motivation, other commitments and medical reasons as the main reasons for drop out across all trials.

### Adverse events

Although only half the studies reported on adverse events, most were non-cardiac related. No cardiac events reported were related to exercise training, which suggests that exercise and the addition of WPAM does not increase incidences of cardiac events. This is in line with numerous studies that have shown low adverse event rates with CR exercise [23, 77, 78]. The specific effect of WPAM on safety cannot be determined from these studies, as only one reported which group (control or intervention) the participants who suffered an adverse event were in.

### Outcome measures

#### Cardiorespiratory fitness/exercise capacity

Our results showed WPAM with exercise prescription or advice improved CRF to a greater extent than no device with the mean overall difference being 1.65 mL/kg/min. A study by Laukkanen et al. [79], observed a 9% reduction in all-cause mortality in those that increased CRF by 1 mL/kg/min over an 11-year period. Our results were higher than Laukkanen et al. [79] suggesting our results are clinically significant. The qualitative best evidence synthesis we conducted also mirrored the results of the meta-analysis in favour of WPAM and suggests there is moderate evidence to support the use of WPAM with exercise prescription or advice on improving CRF/ physical capacity in Phase 3 CR populations.

To the authors’ knowledge, there appears to be no other systematic reviews that have investigated the effect of WPAM with exercise prescription or advice on change in CRF in any population group. It is therefore difficult to directly compare our results to previous studies. However, two studies compared CRF changes as a result of using mobile phone interventions, rather than WPAM. Direito et al. [80] investigated fitness changes in 51 active, young people. Cardiorespiratory fitness was assessed using the 1-mile run/walk test. Our study results contrasted with the results found by Direito et al. [80] as they reported no significant difference in physical fitness compared to the control group. Similarly, another study by Maddison et al. [81] found peak oxygen uptake did not change as a result of a mobile phone intervention including text messages, websites and video messages. The results of this review contrast with studies based within the healthy population as it showed improvements in CRF and may support the use of WPAM with exercise prescription or advice to improve CRF in the cardiac population.

Few studies have investigated the effect of WPAM with exercise prescription or advice on six-minute walk test distance. We found only one study in our review [53] that used the 6MWT as an outcome measure. Varnfield et al. [53] found both control and intervention groups improved six-minute walk test distance, however there was no significant difference between groups. To the authors’ knowledge, there has only been one other study [82] investigating WPAM that used the 6MWT as an outcome measure and was performed with people diagnosed with heart failure. Evangelista et al. [82] reported that patients who showed improvements in their pedometer scores over 6 months also improved their 6MWT distance when compared with patients whose pedometers reflected minimal change in distance walked. Our findings cannot be directly compared; however, the study suggests that participants who adhere more to WPAM with exercise prescription or advice may increase their functional capacity to a greater extent than those adhering less.

#### Pedometer step count

Although our results did not show a significant total effect increase in step count, 70% of the studies reported significant increases in step counts. The sensitivity analysis which removed one study [67] however, did result in a significant difference in step count. It also changed the heterogeneity from substantial to minimal. The sensitivity analysis also increased the effect size (SMD) from 0.45 to 0.78 indicating a moderate effect size [58]. As previously stated, the sensitivity analysis was carried out due to an identified methodological factor which pre-disposed the results to a poorer outcome. This may explain why VO_2_ peak data supported the hypothesis, whilst daily step count data contradicted the hypothesis, and the results of the other three studies. Our qualitative analysis, which compared the results from seven studies, suggests there is moderate evidence to support the use of WPAM with exercise prescription or advice on improving PA in the maintenance phase of CR.

As there have been no systematic reviews investigating the effect of WPAM on step count in the cardiac population, our results cannot be directly compared to the literature. There have been two recent systematic reviews surrounding the effect of smartphone technology and WPAM on the amount of PA performed in healthy subjects. Bort-Roig et al. [83] found five studies with participant numbers ranging from 12 to 42 that investigated PA duration. All studies used step count as the outcome measure. Of the five studies, four (80%) reported increased step count ranging from 800 to 1,104 more steps/day. The duration of the studies ranged between 2 weeks and 6 months. The second systematic review by Muntaner et al. [84] included 12 publications. They investigated the impact of mobile devices on PA. All participants were healthy subjects. The trials used mobile applications, self-reported questionnaires, accelerometers and pedometers. Half of these (6/12) reported significant increases in PA. However, only two of the studies utilised WPAM. Both studies did not investigate the effect of using a WPAM in improving PA. Both groups used pedometers or accelerometers for outcome measures, rather than an intervention. The results of this review resemble findings from the healthy population and suggest the use of WPAM with exercise prescription or advice with exercise prescription or advice may improve step count in the cardiac population.

#### Intensity/Accelerometry data

There are minimal studies investigating the effect of WPAM with exercise prescription or advice on intensity of exercise. Our results showed 75% of studies which measured intensity found a significant increase in the amount of moderate and moderate-high intensity PA of participants compared to the control group for at least one time point. Our results are similar to that found in the Fitbit group by Cadmus-Bertram et al. [75] who investigated the effect of wearing a Fitbit versus wearing a pedometer. Those who wore a Fitbit increased moderate-vigorous activity by 62 ± 108 mins/week. However, those who wore a pedometer did not significantly increase intensity. A further study by Ayabe et al. [85] who investigated WPAM within a chronic disease population, found after 3 weeks, participants who could monitor their intensity using an accelerometer increased time spent in moderate-vigorous activity significantly more than participants who only wore a pedometer. Another study by Finkelstein et al. [34] found the WPAM group performed significantly more moderate-vigorous activity than the control group at 12 months. However, this was not significant at 6 months and further supports the need for longer duration studies. Our results are similar to that reported previously in the literature and suggests WPAM with exercise prescription or advice with exercise prescription or advice may assist in increasing exercise intensity for people diagnosed with cardiac disease.

#### Sedentary time

Our review identified two studies that investigated the effect of WPAM with exercise prescription or advice on sedentary time in the cardiac population. Both studies found no significant differences in sedentary time between the intervention and control groups. These results are similar to that found by Sloan et al. [86], who investigated the effect WPAM had on sedentary behavior in the healthy population. Sloan et al. [86] reported increases in step counts resulted in a decrease in sedentary time, however there was no significant decrease between groups. It appears sedentary time is not influenced by utilising WPAM with exercise prescription or advice.

#### Psychological measures

Our analysis revealed mixed results relating to the improvement of psychological measures when using WPAM with exercise prescription or advice in the maintenance phase of CR. Half the studies showed some statistical difference between group differences in some categories of the respective outcome measure (EDQ5, DASS 21, Kessler 6,overall quality of life) suggesting there may be an effect, although, the studies used a broad range of different measures to investigate psychological effects. There appears to have been no previous reviews or studies that have explicitly aimed to examine the psychological effects of WPAM in people with CVD. However, Maddison et al. [81] did explore the effect of a mobile phone on changes in self efficacy and quality of life. They reported significant improvements in self efficacy and general health domain of the SF 36. In addition, Thorup et al. [49] found participants who used a pedometer reported increased competence to achieve step goals and feelings of support. Participants also reported improved motivation to exercise. Due to our mixed findings, it is therefore difficult to conclude whether WPAM with exercise prescription or advice improve psychological measures or not which is similar to that found by previous literature.

#### Strengths of the review

The strengths of this review include its methodology and statistical analysis. As previously stated, this review is the only analysis of the effectiveness of WPAM with exercise prescription or advice during the maintenance phase of CR. The review also used strict methodology under PROSPERO registration and PRISMA guidelines. Statistical analysis used a conservative approach to calculating standard deviations and reporting was transparent.

#### Limitations

There were several limitations to this review. Using the PEDro scale, we determined that although approximately one third of studies were of good quality, two thirds were of fair quality. There are several improvements that could be made to all studies to increase the confidence in the results. For example, only one study blinded assessors [73] and only two concealed allocation [52, 53]. Study quality assessed through the PEDro scale numerical rating method does not allow for the individual reporting of significant other bias. There were several significant other biases identified during the appraisal of the studies. This included poor completion rates (22% in the intervention group [73] and 43% in the control group [53]) that may have introduced attrition bias by only analysing participants who finished the trial. Poor female representation [52, 67–73] can influence results by measuring a disproportionate gender sample of the population, therefore the results may not have been representative of the general CR population and may be more relevant to males. Finally, one study [69] used block randomisation that delivered treatments over different times of the year. This study was conducted in Ireland where outside temperatures and daylight hours during seasons vary greatly and may have introduced a significant bias by reducing adherence to exercise.

Another significant limitation of this review is the use of concurrent educational/motivational therapies based on the information that a WPAM gives a participant about their activity levels by all studies. Additionally, some studies prescribed specific exercise interventions along with WPAM. These confounding variables make it difficult to distinguish how much influence the WPAM itself or additional exercise prescription, and/or educational/motivational strategies had on the results. However, this review still provides valuable insight into the potential effects of WPAM in the cardiac population despite uncontrolled, concurrent treatments such as exercise prescription potentially contributing to improvements made to key outcomes.

The studies had low homogeneity in several attributes such as timing, length of study, type and parameter of intervention, as well as and type and parameters of control conditions. This is a because our review used data from studies that had different aims to the review, but still, still collected appropriate data on the use of a WPAM in the maintenance phase of CR. For example, one study’s main aim was to evaluate the effectiveness of WPAM in a specific sub- group of non-compliant participants that were up to 1 year post cardiac incident [71]. In particular, the varying commencement of intervention (end of phase 2 or phase 3) may have potentially influenced the results.

Outcome measures used were also a significant source of heterogeneity. Therefore, despite including nine studies in the review, the meta-analysis could only include three studies [67, 70, 73] for VO_2_ peak and four studies [52, 67, 69, 70] for daily step count. These factors imply that although the meta-analyses and review support the hypothesis that WPAM with exercise prescription or advice help to maintain PA in the maintenance phase of CR, these results are based on a small number of studies.

#### Future directions

Additional primary research is needed to investigate the effectiveness of WPAM with exercise prescription or advice on maintaining PA, peak aerobic capacity, intensity of exercise and psychological effects in patients diagnosed with cardiac disease in the maintenance phase of CR. Future studies should attempt to use an attention control group to further strengthen their results by reducing the variables of extra forms of therapy such as specific exercise prescription and motivational therapies. Future studies should blind assessors and incorporate intention to treat analysis to improve quality of trials. With respect to psychological measures, future studies may benefit from investigating general health status (EQ. 5D), psychological distress, (Kessler 6) and Quality of Life index (cardiac version 111), as only these tools showed significant differences between groups in our review. Future studies should focus on good quality methodology, include a large sample number, and utilise consistent outcome measures over a longer follow up period. This would allow analysis of the effects WPAM may have on hospital readmission and mortality rates to be conducted. Comparing effect of WPAM on different genders, specific diagnoses and ensuring reporting which group (control or intervention) participants are in if adverse events occur would be of interest. This would also improve the evidence base for future systematic reviews and strengthening confidence in the results.

## Conclusion

This systematic review and meta-analysis showed that WPAM with exercise prescription or advice significantly improves CRF in the cardiac population to a greater extent than no WPAM. Additionally, our qualitative analysis showed moderate evidence in favour of WPAM for both CRF and step count. The wearing of a WPAM did not change sedentary time. Psychological effects and exercise intensity may potentially be enhanced by using a WPAM. There were no reported cardiac events related to exercise and unrelated medical conditions, lack of motivation and loss of interest were reported as the main reasons for dropping out of trials. Additional longer-term good quality research is required to strengthen these conclusions.

## Data Availability

All data analysed for this review are included in this published article and supplementary material.

## Abbreviations

6MWT: Six-minute walk test
AH: Amanda Hannan
CAP-CR: Care assessment platform
CHD: Coronary heart disease
CI: Confidence interval
CPx: Cardiopulmonary exercise test
CR: Cardiac rehabilitation
CRF: Cardiorespiratory fitness
CVD: Cardiovascular disease
ICD: Implantable cardioverter-defibrillator
IT: Information technology
MD: Mean difference
MH: Michael Harders
PA: Physical activity
PEDro-Scale: Physiotherapy Evidence Database Scale
RCT: Randomised controlled trial
RCTs: Randomised controlled trials
SMD: Standardised mean difference
VO_2_ peak: Peak oxygen uptake
WH: Wayne Hing
WPAM: Wearable PA monitoring devices
